# Supplementary material for: Isoflurane Rescue Schizophrenia-Related Deficits through Parvalbumin-Positive Neurons in the Dentate Gyrus
Source: Biomedicines. 2022 Oct 31;10(11):2759. doi: 10.3390/biomedicines10112759 (PMC9687200; doi:10.3390/biomedicines10112759)
Supplement: Supplementary file 1 [file biomedicines-10-02759-s001.zip › biomedicines-1944576-SM.pdf]

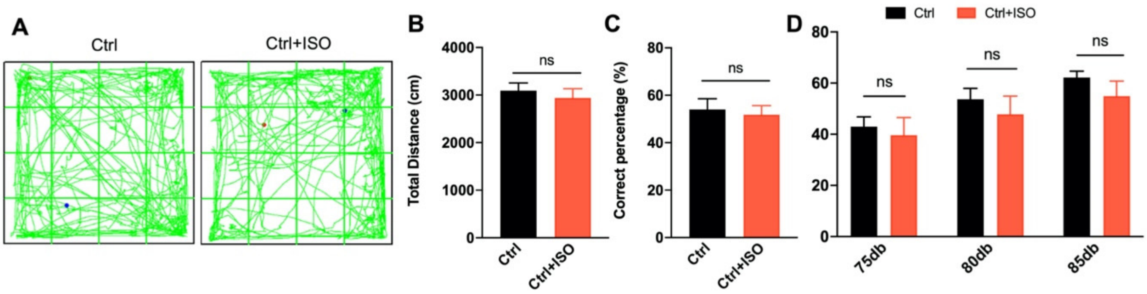

Figure S1 (related to Figure 1). The behavior performance in mice was not affected by Isoflurane inhalation under physiological conditions.

(A) Representative traces of mice in OFT.

(B) Isoflurane treatment didn't affect locomotion activity, revealed by OFT (n=10 in Ctrl, and n=9 in Ctrl+ISO. Unpaired student's t-test, Ctrl vs. Ctrl+ISO, $p = 0.56$).

(C) The working memory was not affacted by isoflurane exposure (n=10 in Ctrl, and n=9 in Ctrl+ISO. Unpaired student's t-test, Ctrl vs. Ctrl+ISO, $p = 0.71$).

(D) Isoflurane didn't disturb the pre-pulse inhibition after five consecutive days exposure (n=10 in Ctrl, and n=9 in Ctrl+ISO. Two-way ANOVA, $F_{(2, 50)} = 0.069$, $p < 0.93$; post hoc test: Ctrl vs. Ctrl+ISO, $p = 0.82$). Data are represented as mean ± SEM.

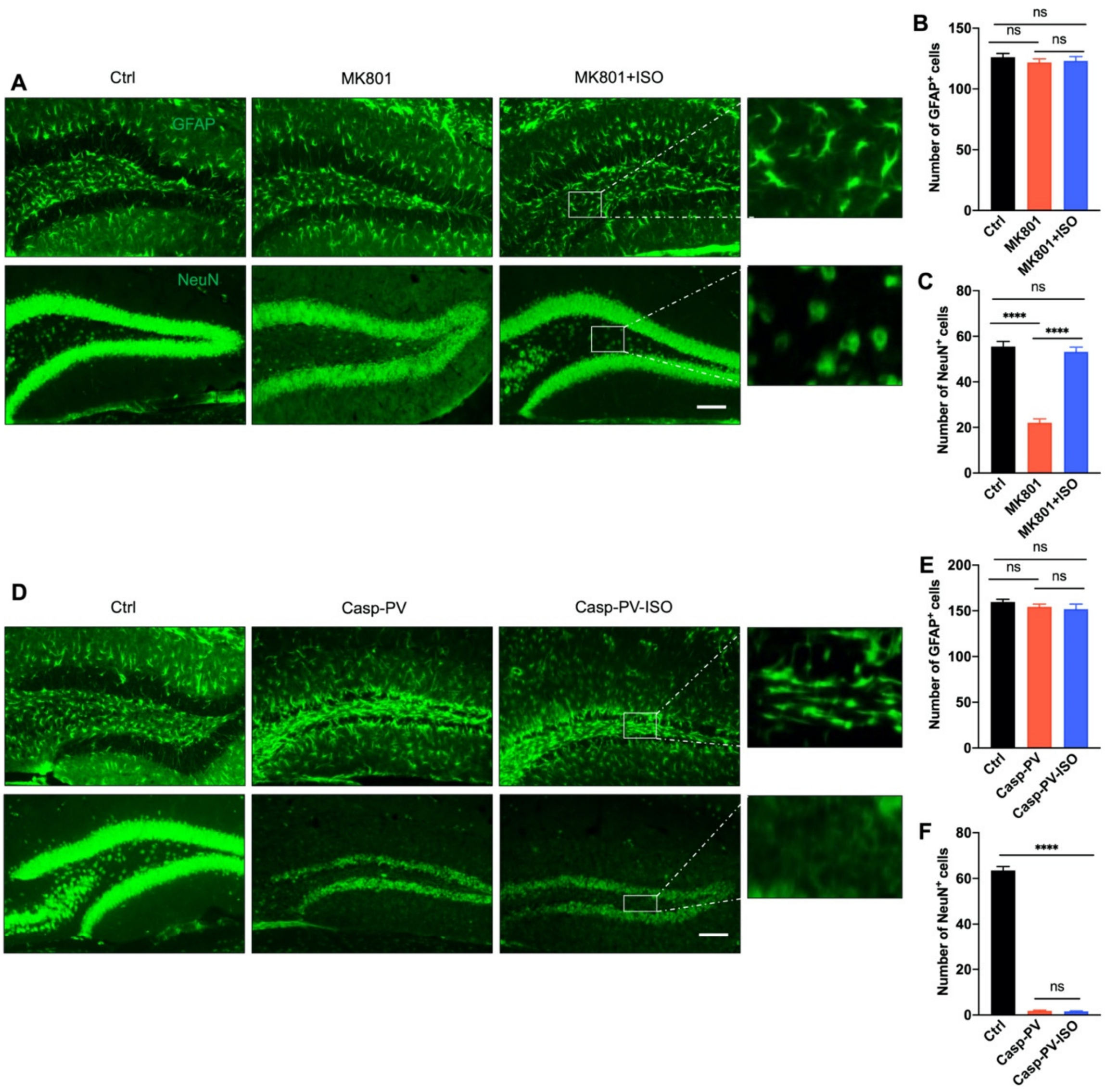

Figure S2. Isoflurane treatment selectively reverses NSCs instead of glial cells in the DG induced by MK801.
(A) Representative photomicrographs showing $GFAP^+$ and $NeuN^+$ cells in the DG. Top: glial cells (green) labelled by GFAP; Bottom: neuronal cells (green) labelled by NeuN. Scale bar, 100 μm.
(B-C) Quantification data of (A). MK801 induced neuronal cells deficits in the DG instead of GFAP. (Five mice in each group, and five sections were picked and counted in each mouse. One-way ANOVA, $F_{(2, 28)} = 0.46$, $p = 0.63$; post hoc test: Ctrl vs. MK801, $p = 0.33$; MK801 vs. MK801+ISO, $p = 0.76$; Ctrl vs. MK801+ISO, $p = 0.53$ shown on (B). One-way ANOVA, $F_{(2, 27)} = 88.39$, $p < 0.0001$; post hoc test: Ctrl vs. MK801, $p < 0.0001$; MK801 vs. MK801+ISO, $p < 0.0001$; Ctrl vs. MK801+ISO, $p = 0.45$ shown on (C)).
(D) Representative photomicrographs showing $GFAP^+$ and $NeuN^+$ cells in the DG. Top: glial cells (green) labelled by GFAP; Bottom: neuronal cells (green) labelled by NeuN. Scale bar, 100 μm.
(E-F) Quantification data of (D). Neuronal cells were ablated in the DG by caspase-3 whereas $GFAP^+$ were not affected. (Five mice in each group, and three sections were picked and counted in each mouse. One-way ANOVA, $F_{(2, 24)} = 0.99$, $p = 0.38$; post hoc test: Ctrl vs. Casp-PV, $p = 0.22$; Ctrl vs. Casp-PV-ISO, $p = 0.67$; Casp-PV vs. Casp-PV-ISO, $p = 0.29$ shown on (E). One-way ANOVA, $F_{(2, 27)} = 1244$, $p < 0.0001$; post hoc test: Ctrl vs. Casp-PV, $p < 0.0001$; Ctrl vs. Casp-PV-ISO, $p < 0.0001$; Casp-PV vs. Casp-PV-ISO, $p = 0.36$ shown on (F). Data are represented as mean ± SEM, $****p < 0.0001$).
